# Diagnostic Reference Levels for Dental Cone Beam Computed Tomography: A Critical Appraisal of Global Progress and Remaining Gaps

**DOI:** 10.7759/cureus.114718

**Published:** 2026-08-18

**Authors:** Danial Aminaei, Asal Ozlati, Daniela Pita de Melo

**Affiliations:** 1 Schulich School of Medicine and Dentistry, Western University, London, CAN; 2 College of Dentistry, University of Saskatchewan, Saskatoon, CAN

**Keywords:** cone beam computed tomography, dental cbct, diagnostic reference levels, dose-area product, patient radiation safety, radiation dose optimization

## Abstract

Cone beam computed tomography (CBCT) is now firmly established in dental and maxillofacial practice, providing three-dimensional visualization for implant planning, endodontics, orthodontics, and oral surgery. However, the expansion of the use of dental CBCT units worldwide has outpaced efforts to standardize and optimize radiation doses delivered to patients. Diagnostic reference levels (DRLs) are meant to fill this gap, acting as benchmarks that flag facilities where doses may need investigation or optimization. Despite this well-established regulatory concept, uptake of DRLs for dental CBCT varies widely from one country to the next.

In this review, we examine where things currently stand globally: the regulatory basis for DRLs, the dose metrics and classification schemes different countries rely on, the DRL values reported in national documents and peer-reviewed literature, and the methodological inconsistencies that make cross-country comparison difficult. Only a small number of countries, mostly in Europe and Japan, have published official national DRLs for dental CBCT. Even among these, there is substantial variation in the dose descriptor used (dose-area product being the most common), in whether classification is based on field of view or clinical indication, and in the resulting DRL values themselves. Perhaps most notably, only one country has established pediatric-specific DRLs for dental CBCT.

We also consider the barriers standing in the way of wider adoption: uneven regulatory infrastructure between countries, a lack of standardized dosimetric protocols for CBCT, limited participation in national dose surveys, and the particular difficulty of overseeing CBCT use in private dental practices that fall outside conventional radiology governance. Moving forward, harmonizing dose metrics internationally, expanding multinational surveys, integrating automated dose-tracking tools, and prioritizing pediatric dose optimization will all be important. Building a stronger global framework for dental CBCT DRLs is essential to keeping pace with an imaging modality that continues to grow in clinical utilization.

## Introduction and background

The introduction of cone beam computed tomography (CBCT) into dental practice has transformed diagnostic imaging of the oral and maxillofacial region. Unlike conventional two-dimensional dental radiographs, which flatten the jaws into a single projection, CBCT provides volumetric three-dimensional datasets that enable clinicians to evaluate intricate anatomical relationships with high spatial resolution [1-2]. Applications now span most dental specialties, including implant planning, assessment of impacted teeth, endodontic assessment, orthodontic analysis, temporomandibular joint assessment, and diagnosis of dentoalveolar pathology [1-3]. The technology has become widely accessible, with CBCT units installed not only in hospital radiology departments and academic centers but also in private dental and community clinics [4].

This extensive adoption, however, has introduced important challenges for radiation protection. CBCT generally delivers higher radiation doses than conventional dental radiography [1,5-6]. Published adult effective doses span approximately 5 to 1,073 microsieverts (µSv), depending on the scanner model, field of view (FOV), exposure parameters, and clinical protocol [5]. By comparison, a panoramic radiograph typically delivers an effective dose of approximately 4-30 µSv [1,6]. A large-FOV or high-dose CBCT protocol can therefore deliver many times the dose of a panoramic examination, and protocol choices on the same machine can produce several-fold dose differences [1,5]. Furthermore, some dental CBCT examinations are performed on children and adolescents, who are more radiosensitive and have a longer remaining lifetime in which radiation-induced effects could manifest [1,7].

Dental CBCT dose can be described using several measures. Effective dose, expressed in µSv, weights organ doses according to tissue sensitivity. It is useful for broad comparisons of stochastic detriment among exposure types or populations, but it is modelled rather than measured directly from a scanner and is not an individualized estimate of patient risk [5,8]. DAP, also termed PKA, is expressed in milligray-square centimeters (mGy·cm²). It represents the integral of air kerma across the X-ray beam area [1,8]. DAP is a measurable equipment-output quantity, not a patient-risk quantity, and is displayed or recorded by many modern CBCT units [1,8]. Because it can be captured routinely at the console, most dental CBCT DRLs use it [1,8]. FOV is the physical volume irradiated during the scan, usually described by its diameter and height in centimeters. All other factors being equal, larger FOVs irradiate more tissue and tend to have higher DAP [1,4-5].

Diagnostic reference levels (DRLs) represent the key international tool for controlling and optimizing patient radiation doses in medical imaging. The concept was introduced by the International Commission on Radiological Protection (ICRP) in Publication 73 in 1996 and was further refined in Publication 135 in 2017 [8,9]. A DRL is defined as the value of a dose quantity that is not expected to be exceeded for standard procedures when good, normal practice regarding diagnostic and technical performance is applied [8]. Importantly, DRLs are not dose limits; rather, they function as investigation levels. When a facility's typical dose consistently exceeds the relevant DRL, this signals the need to review protocols and optimize practice [8-9]. The recommended approach for establishing national DRLs entails conducting surveys across multiple facilities and setting the DRL value at the 75th percentile of the distribution of median facility doses [8].

Despite a well-established regulatory framework and a clear clinical rationale for DRLs, their adoption for dental CBCT has lagged behind that of other imaging modalities [4]. The European Council Directive 2013/59/Euratom mandates the establishment and use of DRLs across all Member States, including for dental exposures [10]. Yet, according to the most recent international surveys, most countries worldwide have not established official DRLs for dental CBCT [4].

Although the regulatory mandate is clear, several questions remain unsettled in the current literature, and these inconsistencies motivate the present review. First, DAP is the most widely used dose descriptor, but the same DAP on two machines with different beam energies and filtration can correspond to different organ and effective doses, and no consensus correction is routinely applied [1,4]. Second, countries are divided between classifying DRLs by FOV size and by clinical indication, and the resulting categories may not be directly comparable [4]. Third, it is unclear how much of the several-fold spread in reported national values reflects real differences in clinical practice rather than differences in survey methodology, sampling, and statistical derivation [4]. Fourth, pediatric-specific national DRLs for dental CBCT remain uncommon: only the United Kingdom and South Korea were identified by the March 2026 search cutoff, despite the long-standing use of age- or weight-stratified pediatric DRLs in medical CT [4,8,11]. Fifth, the availability of usable dose-reference data for the large and expanding CBCT markets of Asia, the Middle East, Africa, and Latin America has not been consistently described [1,4].

Accordingly, the objectives of this review are: (1) to describe the regulatory and conceptual foundations of DRLs as they apply specifically to dental CBCT; (2) to compare the FOV-based and indication-based classification approaches and their implications; (3) to consolidate the published national and regional DRL values into a single comparative summary, including data from under-represented regions; (4) to appraise critically the methodological heterogeneity that limits cross-country comparison; and (5) to identify the principal gaps, with particular attention to pediatric patients, and propose priorities for future work.

## Review

Methodology

Study Design

This narrative review examines literature on dental CBCT DRLs, emphasizing radiation dose optimization, patient safety, approaches to DRL establishment, and methodological differences across countries and regions.

Literature Search

Relevant English-language literature published between 1996 and March 2026 was identified primarily through PubMed. Search terms included combinations of “diagnostic reference level,” “DRL,” “cone beam computed tomography,” “CBCT,” “dental,” “dentomaxillofacial,” “radiation dose,” “dose-area product,” “dose optimization,” “pediatric dental CBCT,” and “radiation protection.”

Relevant governmental, regulatory, and professional publications were also identified from organizations including the International Commission on Radiological Protection (ICRP), International Atomic Energy Agency (IAEA), European Commission, UK Health Security Agency and its predecessor Public Health England, National Council on Radiation Protection and Measurements (NCRP), and European Federation of Organizations for Medical Physics (EFOMP). Reference lists of relevant publications were reviewed to identify additional sources.

Literature was selected based on its relevance to dental and maxillofacial CBCT DRLs, radiation dose assessment, dose optimization, DRL methodology, and pediatric radiation protection. Publications concerning CBCT applications outside the dental and maxillofacial setting were not considered.

Narrative Synthesis

The identified literature was synthesized narratively. Particular attention was given to reported DRL values, dose descriptors, approaches to DRL classification, methods used to establish reference values, and differences between adult and pediatric populations. Published national and regional DRL values were summarized descriptively in Table 1 to facilitate comparison.

**Table 1:** Selected national, regional, and local diagnostic reference levels and reference dose data for dental cone beam computed tomography. Values derived using different classification schemes, sampling frames, measurement methods, and percentile derivations are not directly comparable and should not be read as a ranking of national performance [4,8,19]. In particular, the Sri Lankan values are medians of pooled patient distributions rather than 75th percentiles of facility median values [24]. FOV, field of view; DRL, diagnostic reference level; PKA, air kerma-area product; IAEA, International Atomic Energy Agency; NCRP, National Council on Radiation Protection and Measurements

Country/region	Status	Classification and adult value(s) (mGy·cm²)	Pediatric value (mGy·cm²)	Comment/reference
Germany	Official national DRL	FOV-based; FOV ≤25 cm²: 500; FOV >25 cm²: 1,000	Not established	Two FOV categories [4]
Japan	Official national DRL	FOV-based; <40 cm²: 841; 40-100 cm²: 1,670; >100 cm²: 1960	Not established	Values increase with FOV category [4]
Sweden	Official national DRL	Indication-based; single jaw quadrant: 200; entire upper and lower jaw: 700	Not established	Clinical-region categories [4]
Finland	Official national DRL	Indication-based; single implant: 360; third molar: 380; endodontic/periapical assessment: 550; paranasal sinus: 1150	Not established	Task-specific national values reported in the international appraisal [4]
Switzerland	Official national DRL	Indication-based; wisdom tooth 662; single implant 683; tooth-position anomaly 542; dentoalveolar pathology 569; endodontics 639	Not established	227 of 612 installations responded (37%); possible selection bias [20]
United Kingdom	Official national DRL	Maxillary molar implant: 265	Impacted maxillary canine, child aged about 12 years: 170	National DRLs adopted in 2019; report published in 2020 [29]
Ireland and Italy	Official national DRLs	Maxillary molar implant: 265 (adopted UK value)	Not established	National adoption reported by international survey [4]
Greece	Official national DRL	Values reported in national documentation	Not established	Government-gazette framework reported in 2025 [4]
Estonia	Nationally proposed DRL	One to two implants, FOV <40 cm²: 440	Not established	Described as a proposed national value [4]
South Korea	Official national DRLs	All dental CBCT: 1856	All dental CBCT: 1350	National 2023 survey; private clinics/hospitals had about twice the dose of university dental hospitals [21]
Central and Eastern Europe (IAEA; nine countries)	Regional survey; not formally adopted nationally	Third-quartile PKA: 1000	Third-quartile PKA: 740	27 units and 325 patients; pediatric data from four institutions [22]
United Arab Emirates	Single-scanner typical value	Median PKA: 1000	Not reported	340 adult patients; not a national DRL [23]
Sri Lanka	Multicenter initial reference data; not nationally adopted	Pooled medians by indication: pathology 1013; implant planning 1307; presurgery 1266; sinus/nasal pathology, TMD, or facial trauma 1585	Not separately reported	1162 retrospective scans at four institutions; pooled patient medians, not facility-median 75th percentiles [24]
Iran (Tabriz region)	Local DRLs	Indication-based; implant 341; lesion 387; sinus 342	Not reported	196 patients; single-region sampling frame [25]
India (Tamil Nadu)	Regional proposed DRL	Routine adult CBCT PKA: 532	Not reported	Third quartile from measurements on 10 CBCT units [26]
United States and Canada	No identified national dental CBCT DRL	Not established	Not established	NCRP Report No. 177 provides general US guidance [27]
Africa, Latin America, and much of South and Southeast Asia	No identified national dental CBCT DRL	Not established	Not established	Evidence remains sparse; regional Tamil Nadu data are listed separately [4,26,28]

Differences in methodology, equipment, exposure protocols, field of view, clinical indications, patient populations, and dose descriptors were considered when interpreting reported values. The synthesis was used to identify common approaches, limitations in cross-country comparison, gaps in the available evidence, and priorities for future research and dose optimization.

Regulatory and conceptual foundations of DRLs for dental CBCT

Origins of the DRL Concept

The rationale for DRLs comes from a consistent observation in radiation protection: for a given imaging procedure, dose may vary considerably among facilities, and much of this variation is not explained by patient or clinical factors [8-9]. Dose surveys have shown that facilities at the high end of dose distributions can often reduce exposure while retaining adequate diagnostic performance, indicating substantial potential for optimization [8]. ICRP also recommends reviewing DRLs at intervals of approximately three to five years so that they continue to reflect changes in technology and practice [8].

Why Dental CBCT Poses Distinct Problems

Dental CBCT poses three problems for DRL establishment that do not arise, or arise less acutely, in conventional computed tomography (CT) or general radiography [1,4].

First, conventional CT dose descriptors such as computed tomography dose index (CTDI) are often unsuitable or not routinely available for dental CBCT because cone-beam geometry, partial rotations, beam widths, and detector configurations differ from those of multidetector CT [1,4]. DAP is therefore the recommended practical quantity for dental CBCT DRLs. It can be measured with a calibrated transmission ionization chamber or obtained from a verified console display [1,12,13]. Its relationship to organ or effective dose depends on beam energy, filtration, FOV geometry, and patient anatomy, which complicates direct comparisons among CBCT systems [4,14].

Second, many CBCT units are operated in dental practices and oral surgery clinics that may have less routine access to medical physics support, quality-assurance infrastructure, or automated dose-monitoring systems than hospital radiology departments [4,15]. This can make national surveys and systematic collection of dosimetric data more difficult.

Third, there is a wide variety of CBCT systems on the market, each featuring multiple FOV sizes, voxel resolutions, tube voltage and current settings, and proprietary image reconstruction methods [1,16]. This makes it difficult to characterize them all within a single DRL framework [1,16]. A recent European survey of clinical practice illustrates how far this heterogeneity extends into day-to-day use: among European Academy of DentoMaxilloFacial Radiology members presented with identical diagnostic scenarios, exposure parameters varied widely between clinicians facing the same clinical question, even though dose-area product was registered by around 90% of respondents [17]. Dose variation in dental CBCT is thus not only a between-country and between-scanner phenomenon but a between-operator one, which is precisely the kind of unexplained variation the DRL mechanism exists to address [8,17].

European Regulatory Drivers

European Radiation Protection Report RP 162 set equipment-performance acceptability and suspension criteria that include dental CBCT; failure to meet a suspension criterion calls for corrective action before continued clinical use [18]. These equipment criteria are distinct from patient-dose DRLs, which function as investigation levels for optimization rather than as authorization thresholds for an individual examination [6,8]. The 2013 Euratom Directive further strengthened the mandate by requiring EU Member States to establish, review, and use DRLs for appropriate radiodiagnostic examinations, including those performed in dental settings [10]. Nevertheless, translating these requirements into practical, evidence-based dental CBCT DRLs has been slow and uneven [4]. 

Approaches to DRL Classification: FOV-Based Versus Indication-Based

Two main approaches have emerged for stratifying dental CBCT DRLs: classification by FOV dimensions and classification by clinical indication. Each approach has clear advantages and limitations, and the choice between them has important consequences for how DRL values are interpreted and applied in clinical practice [4,19].

In the FOV-based approach, DRL values are established for categories defined by the imaging volume, often expressed as the product of FOV diameter and height (in cm²) or as descriptors such as small, medium, and large [4,19]. FOV is an important determinant of patient dose: all other exposure factors being equal, larger FOVs irradiate more tissue and tend to require greater total output [1,4,16]. Germany, for instance, uses FOV categories and reports DRLs of 500 mGy·cm² for FOVs up to 25 cm² and 1,000 mGy·cm² for larger FOVs [4]. Japan reports values of 841 mGy·cm² for FOVs below 40 cm², 1670 mGy·cm² for 40-100 cm², and 1960 mGy·cm² for FOVs above 100 cm² [4]. The FOV-based method offers simplicity and wide applicability because it does not require categorization of the clinical indication. However, it does not account for the fact that the same FOV may be used for different diagnostic tasks requiring different image quality and dose levels [4,19].

The indication-based (or clinical application-based) approach stratifies DRL values by specific diagnostic task, such as implant planning, endodontic evaluation, third molar assessment, or orthodontic analysis [4,19]. This corresponds closely with the ICRP's recommendation in Publication 135 that DRLs should ideally be linked to the clinical task, as the required image quality, and hence the acceptable radiation dose, varies with the diagnostic question [8]. Countries adopting this approach include Switzerland, which established DRLs for five dental indications: wisdom tooth assessment (662 mGy·cm²), single-tooth implant planning (683 mGy·cm²), tooth position anomalies (542 mGy·cm²), pathological dentoalveolar modifications (569 mGy·cm²), and endodontics (639 mGy·cm²) [20]. Sweden reported the lowest indication-based DRL value in the literature at 200 mGy·cm² for imaging a single jaw quadrant [4]. The indication-based method delivers a more clinically meaningful framework, but it requires robust data-collection systems able to link dose information to diagnostic categories and relies on consistent coding of indications across facilities, which is not always achievable in busy dental practices [4].

In practice, some countries use blended approaches, or different studies within the same country use different classification schemes, making comparison harder [4]. The French Institute for Radiological Protection and Nuclear Safety proposed a uniform DAP value of 700 mGy·cm² for three adult indications, single implant, extraction of a single impacted tooth, and endodontics, but this proposal was not a value issued by the European Radiation Protection Report RP 162 and has not been universally adopted [4].

Viewed critically, the FOV-versus-indication debate may be a false dichotomy. Neither axis alone captures the determinants of dose: FOV alone ignores the diagnostic task, while indication alone ignores the fact that two clinicians answering the same diagnostic question may irradiate very different volumes at very different beam energies. The most recent international appraisal accordingly recommends that dental CBCT DRLs be structured along four axes simultaneously: patient group (adult or pediatric), FOV, beam energy, and the intended image quality level (low or high), rather than along one [4]. Adopting such a scheme would come at the cost of requiring larger surveys to populate each stratum, which is precisely the constraint that has kept most countries on a single-axis scheme to date [4]. This tension between clinical fidelity and survey feasibility is the central unresolved design problem in this field.

Current Global Status of Dental CBCT DRLs

The most comprehensive recent assessment of the global status of dental CBCT DRLs surveyed members of the Dental Imaging Special Interest Group of the EFOMP, with responses covering 33 countries, including countries outside Europe [4]. It identified national DRLs or national frameworks in ten countries: Finland, Germany, Ireland, Italy, Greece, the United Kingdom, Sweden, Japan, Switzerland, and Estonia, although Estonia's value was described as proposed [4]. Malta, France, and Denmark were reported to be implementing DRLs at the time of that survey [4]. South Korea subsequently reported national adult and pediatric dental CBCT DRLs based on its 2023 national dose survey, bringing the total national frameworks identified by the March 2026 search to 11 [21]. An important caveat is that the EFOMP survey was distributed through a European medical physics network; countries without an active dental medical physics community may therefore be under-represented, and the resulting map should not be treated as an exhaustive global census [4].

Among countries with national DRLs, substantial variation exists. In the clinical indication-based group, reported values range from 200 mGy·cm² for a single jaw quadrant in Sweden to 1150 mGy·cm² for paranasal sinus imaging in Finland [4]. In the FOV-based group, values range from 500 mGy·cm² for FOVs up to 25 cm² in Germany to 1960 mGy·cm² for FOVs above 100 cm² in Japan [4]. This is close to a fourfold spread within each classification group and almost a tenfold spread across the selected published range. The variation may reflect differences in clinical practice and scanner technology, but also differences in survey timing, sample size, dose measurement, and statistical derivation [4,19]. The contribution of each factor cannot be separated reliably from the available reports because national documentation does not consistently provide scanner-fleet composition, indication mix, and derivation method [4,19]. The values in Table 1 should therefore be interpreted within their own regulatory and methodological context, not as a ranking of national performance [4,19].

Data From Asia and the Middle East

In Asia, South Korea's national dose survey established dental CBCT DRLs of 1856 mGy·cm² for adults and 1350 mGy·cm² for children [21]. These comparatively high values may reflect the inclusion of larger-FOV examinations and the scanner mix [21]. The investigators noted that the updated values were higher than both earlier Korean survey values and those of several other countries, and that private dental clinics and hospitals recorded roughly twice the dose levels of university dental hospitals, an internal gradient that demonstrates the optimization function of DRLs [21]. An IAEA survey of nine Central and Eastern European countries involving 27 CBCT units and 325 patients reported third-quartile PKA values of 1000 mGy·cm² for adults and 740 mGy·cm² for pediatric patients, providing regional reference data rather than formally adopted national DRLs [22]. The United Arab Emirates reported a single-scanner typical value, while Sri Lanka reported multicenter pooled patient values intended as initial benchmarks; neither constituted an adopted national DRL framework [23-24].

More recently, a regional study from Tabriz, Iran, derived indication-based local DRLs from 196 patients undergoing common dental CBCT procedures, reporting 341 mGy·cm² for implant examinations, 387 mGy·cm² for lesion assessment, and 342 mGy·cm² for sinus imaging [25]. These values sit at the low end of the selected international range and below the Swiss indication-based values for broadly comparable tasks [20,25]. Whether that difference reflects optimization, a scanner fleet weighted toward small-FOV units, or the single-region sampling frame cannot be determined from the published data [25]; the authors appropriately present them as local rather than national reference levels. Outside Europe and the national programs of Japan and South Korea, much of the available evidence remains regional, institutional, or based on a small number of scanners [4,21-26].

Regions Without National DRL Frameworks

The United States and Canada were not among the countries with identified national dental CBCT DRLs in the international assessment [4]. The United States relies on the NCRP Report No. 177 for general dental radiation protection guidance, but has not established national DRLs specifically for dental CBCT [27]. No national dental CBCT DRLs were identified for countries in the Middle East, Southeast Asia, Latin America, or Africa, highlighting a substantial gap in the available framework [4].

The African situation deserves specific comment because dental CBCT DRLs are absent and diagnostic-radiology DRL evidence remains sparse across much of the continent [4,28]; a recent systematic-review protocol was framed explicitly around this paucity of African data despite continuing acquisition of advanced imaging equipment [28]. South Asia has no identified national dental CBCT DRL, but it is not devoid of multifacility evidence: a Tamil Nadu study measured routine adult exposures on 10 CBCT units and proposed a regional third-quartile PKA of 532 mGy·cm² [26]. This regional result does not substitute for a nationally representative patient survey, but it provides a concrete starting point for Indian dose optimization. Latin America is similarly unrepresented in the identified national dental CBCT DRL literature [4]. In the absence of local reference data, practitioners may have no suitable benchmark for their scanner fleet and indication mix [4]. Establishing a national DRL requires a regulator able to commission surveys, medical physics support to perform and validate measurements, and a reporting system capable of sustaining periodic review [8,12,13]; gaps in that chain plausibly contribute to the geographic pattern observed.

The Key Gap: Pediatric DRLs for Dental CBCT

One major concern in the current literature is the lack of pediatric-specific DRLs for dental CBCT [4,21-22,29]. Children constitute a significant proportion of dental CBCT patients, particularly for orthodontic evaluation, assessment of impacted and supernumerary teeth, and pre-surgical planning for cleft palate and other developmental conditions [1,6]. Children have higher radiosensitivity and longer life spans during which radiation-induced health effects could potentially manifest, making dose optimization especially important [7].

The EFOMP survey identified the United Kingdom as the only country in its sample with an established pediatric-specific dental CBCT DRL [4]. The UK national framework includes a 170 mGy·cm² DRL for localization of an impacted maxillary canine in a child aged approximately 12 years [29]. South Korea subsequently established a national pediatric CBCT DRL of 1350 mGy·cm² based on its 2023 national dose survey [21]. A separate IAEA survey of Central and Eastern Europe reported a regional pediatric third-quartile value of 740 mGy·cm², but this was not a formally adopted national DRL [22]. Thus, by the March 2026 cutoff, national pediatric dental CBCT DRLs were identified in two countries, the United Kingdom and South Korea, while the IAEA value remained regional [4,21-22,29]. These sparse data contrast with the established use of age- or weight-stratified DRLs for pediatric medical CT in many countries [8,11].

ICRP Publication 135 recommends weight bands for pediatric DRLs involving the trunk and emphasizes adapting imaging procedures for children [8]. Applying that guidance to dental CBCT is not straightforward because ICRP recommends age groupings for head examinations, whereas dental CBCT protocols must also account for the anatomical region and diagnostic task [1,8]. Nevertheless, scan parameters should be adapted for pediatric patients, and reference values are needed to benchmark that practice [1,6-7]. Encouragingly, European survey data indicate that many clinicians already adjust pediatric protocols: approximately 70% reported reducing FOV, 67% reducing tube current, and 59% reducing exposure time [17]. The remaining problem is the scarcity of national benchmarks against which those reductions can be evaluated [4,21-22,29]. Pediatric DRLs would convert the general expectation that children receive less exposure into auditable, indication-linked optimization targets.

Methodological Challenges and Barriers to Harmonization

Multiple practical and methodological barriers make it difficult to establish and standardize dental CBCT DRLs globally. Four in particular recur across the literature [4,15,19-20,22].

First, the lack of a universally harmonized dose metric and stratification scheme is a fundamental obstacle [4]. DAP is the most commonly reported equipment-output descriptor for dental CBCT, but its interpretation is complicated by differences in beam energy, which commonly spans approximately 60-120 kV, filtration, and FOV geometry among systems [1,4]. The same DAP from two units may correspond to different organ or effective doses because the X-ray spectrum and irradiated anatomy differ [4,14]. One proposed approach is to stratify DRLs by beam half-value layer (HVL), with a suggested 6 mm Al boundary between lower- and higher-energy protocols, but this has not been widely validated [4].

Second, conducting representative national dose surveys for dental CBCT is operationally difficult [4,15,20]. Unlike hospital-based imaging modalities, where dose data may be collected through radiology information systems and picture archiving and communication systems (PACS), dental CBCT units in private practices frequently lack such infrastructure [4,15]. Survey participation is typically voluntary, and response rates may be low [4,20]. The Swiss national survey received data from 227 of 612 CBCT installations (37.1%; reported by the authors as a 38% response rate) [20]. This raises the possibility of selection bias because participating practices may not represent the wider population [20]. ICRP guidance indicates that initial surveys in smaller countries should ideally include approximately 30-50% of installations, a benchmark that may be difficult to achieve [8,22].

Third, significant differences in how data are reported across studies make meaningful international comparisons difficult. Some studies report DRLs stratified by FOV category, others by clinical indication; some report PKA measured directly, others calculate it from displayed scanner parameters; and the statistical methods for deriving the 75th percentile value differ between studies that use individual patient-level data versus those that use facility-level median data [4,19]. This last distinction is not a technicality: deriving a percentile from pooled patient data allows high-volume facilities to dominate the distribution, whereas the facility-median approach recommended by ICRP weights each facility equally, and the two can yield materially different reference values from the same underlying dataset [8,19,24].

Furthermore, CBCT technology continues to evolve, with newer systems offering dose-reduction features such as more sensitive detectors, optimized pulsed exposure, and task-specific low-dose modes [1,30]. DRLs derived from older scanner populations may therefore overestimate doses achievable with current technology, supporting the ICRP recommendation for review at intervals of approximately three to five years [8,30]. Yet technology alone does not guarantee optimization: a 2025 study of 7320 scans at eight Central and Northern European university centers reported DAP values from 34 to 4390 mGy·cm², and dedicated dose-reduction modes were used in only 21% of examinations [31]. Together with the large operator variation reported by European Academy of DentoMaxilloFacial Radiology (EADMFR) members [17], these findings indicate that protocol selection and implementation remain central [17,31].

Fourth, the regulatory and professional environment surrounding dental CBCT varies markedly among countries [4,15]. Some jurisdictions place dental CBCT under national radiation-protection oversight with defined mechanisms for dose surveys and DRL setting. Elsewhere, limited medical physics capacity, fragmented private-practice governance, and the absence of routine dose-reporting systems impede representative surveys [4,15]. The IAEA's nine-country Central and Eastern European study demonstrates the value of coordinated regional work, but it also included only 27 units and pediatric data from four institutions, illustrating the difficulty of building representative datasets [22]. These barriers reinforce one another, which helps explain why isolated initiatives have not yet produced a comparable global picture [4,15,22].

Future directions and recommendations

Addressing the gaps identified in this review requires coordinated action [4,8]. Standardizing dose metrics should be a priority for bodies such as the ICRP, IAEA, and EFOMP because it is a prerequisite for meaningful international comparison [4,8,12,13]. A consensus protocol should specify the dose quantity (usually DAP), measurement and display-verification methods, reporting format, statistical derivation, and classification scheme, ideally incorporating patient group, FOV, beam energy, and required image quality [4,12,13]. The proposal to stratify DRLs by beam HVL to account for spectral differences among CBCT models warrants validation through multicenter phantom and patient studies [4].

Expanding national and multinational dose surveys is essential, especially in regions that lack national dental CBCT DRLs [4,22]. The coordinated IAEA survey in Central and Eastern Europe provides a model that could be adapted for Latin America, Sub-Saharan Africa, and South and Southeast Asia [22]. Such efforts should collect not only dose values but also clinical indications, scanner models and settings, measurement or display-verification methods, and facility-level typical values [4,8]. Where a national survey is not yet feasible, the Emirati, Iranian, and Tamil Nadu studies illustrate intermediate approaches: establishing a defined institutional or regional baseline that can support optimization and later, broader surveys [23,25-26].

Incorporating automated dose tracking and reporting into dental CBCT workflows could facilitate both DRL surveys and continuous local monitoring [4,32]. Many CBCT units display or record DAP, while a published dental CBCT architecture has shown that Digital Imaging and Communications in Medicine (DICOM) Radiation Dose Structured Reports can feed automated cloud-based DRL monitoring [4,32]. Standardized export and validation would allow larger datasets to be collected with less manual transcription, although displayed dose values must still be verified for accuracy [12,13]. The finding that approximately 90% of surveyed EADMFR practitioners already registered DAP suggests that a major remaining obstacle is standardized export, aggregation, and interpretation rather than initial capture alone [17].

Establishing pediatric DRLs for dental CBCT should remain a priority [4,8,11,21-22,29]. This will require dedicated pediatric dose surveys, potentially supplemented by pediatric-sized phantoms to account for differences in anatomy and attenuation [6,8]. Low-dose protocol studies show that substantial reductions can be achieved for defined pediatric tasks while preserving diagnostically acceptable image quality, but such protocol optimization is not equivalent to establishing a population DRL [33]. International collaboration may be valuable because individual centers often perform relatively few pediatric CBCT examinations [4,6-7].

Finally, education and training of dental professionals in radiation protection and dose optimization deserve sustained attention. Training standards and clinical recommendations emphasize justification, protocol selection, and operator competence for dental CBCT [2,34-36]. Incorporating DRL education into dental curricula, continuing professional development, and vendor training could help translate dose benchmarks into routine optimization [4,34-36]. The ALADAIP principle (as low as diagnostically acceptable, indication-oriented, and patient-specific) provides a useful organizing framework by linking dose reduction to the diagnostic task and the individual patient [17,36].

Limitations

Several limitations should be borne in mind when interpreting this review. First, the design is narrative rather than systematic: no formal risk-of-bias instrument or quantitative synthesis was applied. Second, the search was restricted to PubMed together with targeted regulatory and organizational sources; other databases were not searched, and relevant records indexed only in those databases may have been missed. Third, the restriction to English-language records is likely to have excluded national DRL documentation published only in national languages, which is a material limitation given that DRLs are promulgated as national regulatory instruments. Finally, much of the global picture presented here rests on a single international survey [4], whose sampling frame consisted of members of a European medical physics special interest group and therefore under-represents regions without an established dental medical physics community.

## Conclusions

Diagnostic reference levels are an internationally endorsed means of optimizing patient radiation protection in medical imaging, but their application to dental CBCT remains incomplete and inconsistent. Only a small number of countries, concentrated in Europe and East Asia, have established national dental CBCT DRLs; most countries, including large markets with substantial CBCT use, lack a formal national framework. Differences in classification, measurement, sampling, and statistical derivation hamper international comparison. The selected published values span nearly an order of magnitude, but the available data do not allow this spread to be attributed confidently to practice rather than method, a finding that itself strongly supports a consensus survey protocol. Pediatric national DRLs remain particularly rare: the United Kingdom and South Korea were the only countries identified by the March 2026 cutoff, with additional regional data from the IAEA Central and Eastern European survey. Regional studies such as the Tamil Nadu survey show that evidence is emerging in under-represented areas, but it does not replace nationally representative surveillance. Progress requires standardized dosimetry and reporting, broader national and multinational surveys, validated automated dose tracking, pediatric optimization, and sustained professional education. As dental CBCT use grows, strengthening the DRL framework is a practical clinical requirement for patient protection.

## References

[1] Kaasalainen T, Ekholm M, Siiskonen T, Kortesniemi M (2021). Dental cone beam CT: an updated review. Phys Med.

[2] Horner K, Islam M, Flygare L, Tsiklakis K, Whaites E (2009). Basic principles for use of dental cone beam computed tomography: consensus guidelines of the European Academy of Dental and Maxillofacial Radiology. Dentomaxillofac Radiol.

[3] Jacobs R, Salmon B, Codari M, Hassan B, Bornstein MM (2018). Cone beam computed tomography in implant dentistry: recommendations for clinical use. BMC Oral Health.

[4] Trindade H, Toivo Mark EC, Camilleri ML, Tsaggari E, Gilligan P, Pauwels R (2025). Diagnostic reference levels for dental cone-beam computed tomography: current state and way forward. Phys Med.

[5] Ludlow JB, Timothy R, Walker C, Hunter R, Benavides E, Samuelson DB, Scheske MJ (2015). Effective dose of dental CBCT-a meta analysis of published data and additional data for nine CBCT units. Dentomaxillofac Radiol.

[6] European Commission, Directorate-General for Energy (2012). Radiation Protection No. 172: cone beam CT for dental and maxillofacial radiology - evidence-based guidelines (SEDENTEXCT project). Radiation Protection No. 172: Cone Beam CT for Dental and Maxillofacial Radiology - Evidence-Based Guidelines (SEDENTEXCT Project).

[7] (2007). The 2007 Recommendations of the International Commission on Radiological Protection. ICRP publication 103. Ann ICRP.

[8] Vañó E, Miller DL, Martin CJ (2017). ICRP publication 135: Diagnostic reference levels in medical imaging. Ann ICRP.

[9] International Commission on Radiological Protection (1996). Radiological protection and safety in medicine. Ann ICRP.

[10] European Council (2026). Council of the European Union: Council Directive 2013/59/Euratom of 5 December 2013 laying down basic safety standards for protection against the dangers arising from exposure to ionising radiation, and repealing Directives 89/618/Euratom, 90/641/Euratom, 96/29/Euratom, 97/43/Euratom and 2003/122/Euratom. Off J Eur Union.

[11] Priyanka Priyanka, Kadavigere R, Sukumar S, Pendem S (2021). Diagnostic reference levels for computed tomography examinations in pediatric population - a systematic review. J Cancer Res Ther.

[12] International Atomic Energy Agency (2007). Dosimetry in diagnostic radiology: an international code of practice. Technical Reports Series No. 457. Dosimetry in Diagnostic Radiology: An International Code of Practice. Technical Reports Series No. 457.

[13] de Las Heras Gala H, Torresin A, Dasu A (2017). Quality Control in Cone-Beam Computed Tomography (CBCT): EFOMP-ESTRO-IAEA Protocol. Quality Control in Cone-Beam Computed Tomography (CBCT): EFOMP-ESTRO-IAEA Protocol.

[14] Rottke D, Patzelt S, Poxleitner P, Schulze D (2013). Effective dose span of ten different cone beam CT devices. Dentomaxillofac Radiol.

[15] Vassileva J, Stoyanov D (2010). Quality control and patient dosimetry in dental cone beam CT. Radiat Prot Dosimetry.

[16] Pauwels R, Beinsberger J, Collaert B (2012). Effective dose range for dental cone beam computed tomography scanners. Eur J Radiol.

[17] Fakhtei S, Hoogeveen R, Berkhout E (2026). Dose optimization in CBCT in dentistry: a survey among EADMFR members. Dentomaxillofac Radiol.

[18] European Commission, Directorate-General for Energy (2012). Radiation Protection No. 162: criteria for acceptability of medical radiological equipment used in diagnostic radiology, nuclear medicine and radiotherapy. Radiation Protection No. 162: Criteria for Acceptability of Medical Radiological Equipment Used in Diagnostic Radiology, Nuclear Medicine and Radiotherapy.

[19] McGuigan MB, Duncan HF, Horner K (2018). An analysis of effective dose optimization and its impact on image quality and diagnostic efficacy relating to dental cone beam computed tomography (CBCT). Swiss Dent J.

[20] Deleu M, Dagassan D, Berg I (2020). Establishment of national diagnostic reference levels in dental cone beam computed tomography in Switzerland. Dentomaxillofac Radiol.

[21] Kim JE, Yeom HG, Hwang JJ (2025). National dose survey and discussion on establishing diagnostic reference levels for dental imaging in Korea. Dentomaxillofac Radiol.

[22] Beganović A, Ciraj-Bjelac O, Dyakov I (2019). IAEA survey of dental cone beam computed tomography practice and related patient exposure in nine Central and Eastern European countries. Dentomaxillofac Radiol.

[23] Abuzaid MM, Elshami W, Jayachandran D, Korappil N, Tekin HO (2022). Establishment of diagnostic reference levels in cone beam computed tomography scans in the United Arab Emirates. Tomography.

[24] Nissanka M, Satharasinghe D, Jeyasugiththan J (2024). Radiation dose assessment during dental cone beam computed tomography procedures in Sri Lanka towards establishing a dose reference level. Radiat Prot Dosimetry.

[25] Mardfar S, Fazel Ghaziyani M, Saeedi Vahdat A, Khezerloo D (2025). Establishment of diagnostic reference levels for dental cone beam computed tomography in Tabriz, Iran. Radiat Prot Dosimetry.

[26] Jose A, Kumar AS, Govindarajan KN, Sharma SD (2022). Assessment of regional diagnostic reference levels in dental radiography in Tamil Nadu. J Med Phys.

[27] National Council on Radiation Protection and Measurements (2019). Radiation protection in dentistry and oral & maxillofacial imaging. NCRP Report No. 177. Radiation Protection in Dentistry and Oral & Maxillofacial Imaging. NCRP Report No. 177.

[28] Adekanmi AJ, Akinlade BI, Adedoyin OA (2025). Diagnostic reference levels in Africa: a protocol for systematic review and meta-analysis. Afr Health Sci.

[29] Holroyd JR, Smith JRH, Edyvean S (2020). Dose to Patients From Dental Radiographic X-Ray Imaging Procedures in the UK - 2017 Review. PHE-CRCE-59. PHE-CRCE-59. Public Health England, London.

[30] Kiljunen T, Kaasalainen T, Suomalainen A, Kortesniemi M (2015). Dental cone beam CT: a review. Phys Med.

[31] Schulze R, Klingler S, Biel P (2025). Cone beam computed tomography - frequency and exposure settings at university (dental) hospitals in Central and Northern Europe. Eur J Radiol.

[32] Esmaeilyfard R, Samanipour A, Paknahad M (2021). A cloud-fog software architecture for dental CBCT dose monitoring using the DICOM structured report: automated establishment of DRL. Phys Med.

[33] Hidalgo Rivas JA, Horner K, Thiruvenkatachari B, Davies J, Theodorakou C (2015). Development of a low-dose protocol for cone beam CT examinations of the anterior maxilla in children. Br J Radiol.

[34] Brown J, Jacobs R, Levring Jäghagen E, Lindh C, Baksi G, Schulze D, Schulze R (2014). Basic training requirements for the use of dental CBCT by dentists: a position paper prepared by the European Academy of DentoMaxilloFacial Radiology. Dentomaxillofac Radiol.

[35] Tsapaki V (2017). Radiation protection in dental radiology - recent advances and future directions. Phys Med.

[36] Benavides E, Krecioch JR, Connolly RT (2024). Optimizing radiation safety in dentistry: clinical recommendations and regulatory considerations. J Am Dent Assoc.

